# Interesting Cytokine Profile Caused by Clinical Strains of *Pseudomonas aeruginosa* MDR Carrying the exoU Gene

**DOI:** 10.1155/2024/2748842

**Published:** 2024-06-30

**Authors:** Nallely S. Badillo-Larios, Edgar Alejandro Turrubiartes-Martínez, Esther Layseca-Espinosa, Roberto González-Amaro, Luis Fernando Pérez-González, Perla Niño-Moreno

**Affiliations:** ^1^ Center of Research in Health Sciences and Biomedicine Faculty of Medicine Autonomous University of San Luis Potosi, San Luis Potosi, Mexico; ^2^ Laboratory of Hematology, Faculty of Chemical Sciences Autonomous University of San Luis Potosi, San Luis Potosi, Mexico; ^3^ Faculty of Medicine Autonomous University of San Luis Potosi, San Luis Potosi, Mexico; ^4^ Central Hospital Dr. Ignacio Morones Prieto, San Luis Potosi, Mexico; ^5^ Genetics Laboratory Faculty of Chemical Sciences Autonomous University of San Luis Potosi, San Luis Potosi, Mexico

## Abstract

*Pseudomonas aeruginosa* is an opportunistic pathogen in HAIs with two facets: the most studied is the high rate of antimicrobial resistance, and the less explored is the long list of virulence factors it possesses. This study aimed to characterize the virulence genes carried by strains as well as the profile of cytokines related to inflammation, according to the resistance profile presented. This study aims to identify the virulence factors associated with MDR strains, particularly those resistant to carbapenems, and assess whether there is a cytokine profile that correlates with these characteristics. As methodology species were identified by classical microbiological techniques and confirmed by molecular biology, resistance levels were determined by the minimum inhibitory concentration and identification of MDR strains. Virulence factor genotyping was performed using PCR. In addition, biofilm production was assessed using crystal violet staining. Finally, the strains were cocultured with PBMC, and cell survival and the cytokines IL-1*β*, IL-6, IL-10, IL-8, and TNF-*α* were quantified using flow cytometry. Bacteremia and nosocomial pneumonia in adults are the most frequent types of infection. In the toxigenic aspect, genes corresponding to the type III secretion system were present in at least 50% of cases. In addition, PBMC exposed to strains of four different categories according to their resistance and toxicity showed a differential pattern of cytokine expression, a decrease in IL-10, IL-6, and IL-8, and an over-secretion of IL-1b. In conclusion, the virulence genes showed a differentiated appearance for the two most aggressive exotoxins of T3SS (*exoU* and *exoS*) in multidrug-resistant strains. Moreover, the cytokine profile displays a low expression of cytokines with anti-inflammatory and proinflammatory effects in strains carrying the exoU gene.

## 1. Introduction

*Pseudomonas aeruginosa* is a Gram-negative bacterium and one of the most important pathogens in healthcare-associated infections (HAIs), causing 32,600 infections and 2,700 deaths in the United States in 2017 [1]. Classified as opportunistic pathogens involved in acute and chronic infections, the main reason for this versatility is the large list of virulence determinants. It possesses both intrinsic and acquired resistance to antibiotics and high metabolic flexibility because of its ability to use various carbon sources or electron acceptors [2]. Owing to these mechanisms, *P. aeruginosa* is responsible for several HCAIs, such as pneumonia, surgical infections, bacteremia, and urinary tract infections [3]. The challenge of treating an infection caused by *P. aeruginosa* is why the World Health Organization (WHO) has included it in the “critical” category in its priority list of pathogens that urgently require research and development of new antibiotics [4].

According to a review in 2021, it is estimated that 7.1%–7.3% of HCAIs are caused by this pathogen, and the most common infection is pneumonia, followed by surgical site infection (SSI); however, its prevalence has increased over the last decade [5]. In addition, the available data on the Mexican situation are limited; in 2022, the RHOVE (Red Hospitalaria de Vigilancia Epidemiológica, in Spanish) found *P. aeruginosa* to be the second most common cause of HCAIs in the country [6]. The latest information provided by the IMSS (Instituto Mexicano del Seguro Social, in Spanish) in 2016 points to *P. aeruginosa* as the third cause, just below *Escherichia coli* and *Staphylococcus aureus* [7]. Another report pointed out that Mexico has the highest rate of infection caused by this pathogen [8].

As mentioned above, the complexity of the infections caused by *P. aeruginosa* has two faces: the most studied is the high rate of antimicrobial resistance, especially resistance to carbapenems, with 17%–52% of the cases worldwide [9], and the ultimate concern is multidrug-resistant (MDR) strains, representing 9% of cases in the USA in 2018 [10].

Another issue mentioned above is the ample list of virulence factors possessed by *P. aeruginosa*, some of which are enzymes, exoenzymes, toxins, and the ability to grow as a planktonic community as well as in biofilms [11]. In this respect, the arsenal includes factors that are associated with the bacterial surface and those secreted, which include exotoxin A, phospholipase, alkaline protease, pyoverdine, elastases, pyocyanin, and the type three secretion system injectosome, which can deliver four different toxins in the cellular host [2, 12].

The contribution of each factor to the deterioration of the cellular host is variable, and according to Moradali, environmental stress induces differential genomic expression, forming persistent, resistant, but less virulent phenotypes in chronic illness [2]. In acute infections, virulence factors, such as flagella and pili, are vital because they are involved in motility and initial attachment. The flagellum provides swimming motility in an aqueous environment, is essential for bacterial chemotaxis, and adheres to epithelial cells by binding its flagellum to the glycolipid asialo GM1, which can also initiate the inflammatory response. Pili is an important adhesin. It is also a decisive factor for motility, but it does so through a movement called twitching. Together, these two factors also create a highly coordinated form of motility called swarming, which is useful for semisolid surfaces [12].

*P. aeruginosa* produces lectins that bind with specific sugars in the initial attachment of the bacteria, particularly LecB (also named PII-L), which reduces ciliary beating of the airway epithelium and is linked to biofilm formation, serving as a structural protein in the matrix [13].

*P. aeruginosa* owns the exotoxin A, which is an ADP-ribosyl transferase related to attachment that enters the cell and inhibits host protein synthesis by inactivating eukaryotic elongation factor 2 [14].

Furthermore, this pathogen has several proteolytic enzymes, such as elastase A and B (LasA, LasB), secreted by the type 2 secretor system (T2SS), both capable of degrading host elastin. Specifically, LecB, also named “pseudolysin,” is considered the most abundant protease capable of disrupting epithelial tight junctions [15].

Alkaline protease, also known as “aeruginosin,” secreted by the type 1 secretor system, is a metalloendopeptidase that can damage the epithelium, interfering with fibronectin and laminin [16]. Phospholipase C, a lipolytic enzyme secreted by T2SS, has hemolytic activity capable of destroying the eukaryotic membrane and destabilizing phospholipids and sphingomyelin [17].

Other important factors include the pigments produced by *P. aeruginosa*, such as pyoverdine, pyochelin, and pyocyanin. The first one is associated with the characteristic fluorescent green of the species and is related to the acquisition of iron, which is highly efficient but requires enormous amounts of energy in its production. In most cases, active pyochelin is produced [18]. The other pigment, pyocyanin, which is responsible for the blue-greenish color of the colonies in culture, is a phenazine and secondary metabolite related to the decline in lung function because of its inflammatory effects [19].

Another secretor system that is important to review is the type three secretion system (T3SS), a syringe-like injectosome that can deliver toxins directly into the host cell [20] and has four toxin effectors: ExoY, ExoT, ExoU, and ExoS. The first two were the most abundant and were expressed in more than 89% of the isolates. ExoY is an adenylate cyclase capable of causing cell necrosis, endothelial disruption, and irreversible actin microtubule disassembly [21]. ExoT, the most prevalent of these effectors, has two main functions: acting as an exotoxin with GTPase-activating protein (GAP) and as an adenosine diphosphate-ribosyl transferase (ADPRT), which disrupts epithelial barriers [22]. However, neither of these toxins is thought to be sufficient to establish an infection specifically in the lungs [21, 22].

The two remaining molecules are less common but highly lethal. ExoS is a GAP found in 55%–72% of clinical isolates and is associated with chronic infections, capable of producing cell death and actin cytoskeletal disruption [23]. Finally, ExoU is less frequent (28%–42% of the isolates), it has phospholipase activity with cytotoxic effects that rapidly destroy the cell membranes of mammalian cells, and some studies have found that it is more frequent in isolates with high resistance [24].

This study aimed to identify the virulence factors associated with MDR strains, particularly those resistant to carbapenems, and assess whether there is a cytokine profile that correlates with these characteristics.

## 2. Methods

### 2.1. Bacterial Isolates

A total of 75 isolates of *Pseudomonas aeruginosa* from infections classified as HCAIs were obtained from a tertiary hospital (HCIMP in San Luis Potosi, Mexico) between May 2018 and June 2019 before approval by the Research Committee and the Research Ethics Committee of the HCIMP. Informed consent was obtained from all participants or legal guardians.

Data collected from the medical records of the patients included sex, age, length of hospital stay, and outcome.

### 2.2. Bacterial Identification and Antimicrobial Susceptibility Testing

Species identification and antimicrobial susceptibility testing were performed using an automated VITEK 2 (BioMérieux SA. F-69280 Marcy l'Etoile, France). Additionally, PCR amplification was performed to confirm species identity using the primers described by Spilker et al., including primers Pa-gs for the genus and Pa-ss for the species (16S rDNA) [25].

The susceptibility profiles were confirmed using broth microdilution. Briefly, antibiotics were serially diluted 2-fold in 50 *μ*L of Mueller-Hinton broth, mixed with 50 *μ*L of bacteria at a density of 10^6^ colony-forming units/mL, and incubated for 18 h at 37°C. Antimicrobial susceptibility testing was performed using the following antibiotics: amikacin, cefepime, gentamicin, piperacillin/tazobactam, aztreonam, ciprofloxacin, and meropenem. The results were interpreted according to guidelines recommended by the Clinical and Laboratory Standards Institute [26]. Additionally, isolates were classified as nonmultidrug-resistant (NMDR) or MDR (defined as acquired nonsusceptibility to at least one agent in ≥3 antimicrobial categories).

### 2.3. DNA Extraction

Genomic DNA was extracted using a boiling method as described previously, and one loopful of fresh bacteria (grown overnight on Brain-Heart Infusion agar plates) was collected and suspended in 200 *µ*L of sterile DNase/RNase-free water and incubated at 94 °C for 5 min and −70 °C for 5 min. The bacterial suspension was then centrifuged at 13,000 rpm at 4°C for 3 min, and the supernatant was collected and stored at −20°C.

### 2.4. Virulence Factor Detection

Detection of the following virulence genes was carried out by conventional PCR: type 4 fimbrial biogenesis protein (*pilB*), alginate (*algD*), alkaline protease (*aprA*), elastase (*lasB*), exoenzymes for the type three secretion system (*exoS, exoU, exoT,* and *exoY*), exotoxin A (*toxA*), hemolytic phospholipase C (*plcH*), elastase B (*lasB*), pyoverdine (*pvdA*), a lectin (*lecB*), a phenazine (*phzM*), and flagellin (*flag*). Primers, temperature melting (Tm), and expected amplicons of genes associated with virulence used in this study are listed in Supplementary Table 1. Amplification was carried out in a 25-*μ*L volume containing 23 *μ*L of PCR Master mix (DreamTaq Green PCR master mix, Thermo Scientific), 1 *μ*L of each primer (forward and reverse), 1 *μ*L of template DNA, and nuclease-free water.

### 2.5. Biofilm Production

Biofilm formation was determined using crystal violet staining. Briefly, 200 *µ*L of TSA broth inoculated with the strains at a concentration of 0.5 on the MacFarland scale was dispensed into a 96-well polystyrene microtiter plate and incubated at 37°C for 24 h. After incubation, the contents of each well were removed by gentle tapping and washed four times with 250 *µ*L of phosphate-buffered saline (pH 7.2) to remove free-floating bacteria. Biofilms formed by adherent bacteria were fixed by air drying. Afterward, wells were stained with crystal violet (0.1%) for 10 minutes. The excess stain was washed three times with deionized water, the plates were dried, and the dye bound to the cells was resolubilized with 200 *μ*L of absolute methanol per well. The optical density (OD) of the stained adherent biofilm was measured using an Epoch Microplate Spectrophotometer (Winooski, VT, USA) at a wavelength of 590 nm. The negative control wells contained inoculated sterile broth, and every experiment was performed in triplicate and repeated three times. Biofilm production was interpreted according to Stepanovic et al. using the following criteria: OD > ODc = no biofilm producer, ODc < OD = 2 (ODc) = weak biofilm producer, 2 (ODc) < OD = 4 (ODc) = moderate biofilm producer, 4 (ODc) < OD = strong biofilm producer, and ODc as three standard deviations (SD) above the mean OD of the negative control [27].

### 2.6. Isolation of Peripheral Blood Mononuclear Cells (PBMCs)

Peripheral blood from healthy donors was collected in heparin (with informed consent), and PBMCs were obtained using a standard gradient protocol (Ficoll-Paque™ Plus, Merck KGaA, Darmstadt, Germany). Briefly, blood was diluted with PBS in a 1 : 1 ratio, and 30 mL of the mixture was layered into 10-mL Ficoll-Paque PLUS in 50-mL tubes. The tubes were centrifugated at 1000 × *g* for 20 minutes at 20°C without breakage. Buffy coats were collected, pooled, resuspended in PBS, and centrifuged at 400 × *g* for 20 min without breaking. Cells were washed twice with sterile PBS, and after centrifugation (400 × *g*, 30 min), a layer of PBMCs was obtained and collected, and cells were counted in a Neubauer chamber. Cell viability was evaluated using the trypan blue exclusion method (Sigma-Aldrich, USA). Cells were used only when viability was >98%. Cells were suspended in complete RPMI containing the RPMI 1640 medium supplemented with 10% fetal bovine serum (previously inactivated with heat at 56°C for 30 minutes) and 2 mM L-glutamine.

### 2.7. PBMC: Bacterial Coculture

Sixteen strains recovered from respiratory infections were selected and classified into four groups according to their susceptibility pattern (MDR and non-MDR) and the exotoxin genes detected (*exoU* and *exoS*). A concentration of 1 × 10^6^ CFU/mL with PBMC at a concentration of 1 × 10^6^ CFU/mL was seeded in 1-mL 24-well plates.

PBMC bacterial cocultures were incubated in complete RPMI as mentioned before at 37°C in 5% CO_2_ for 24 h. PBMCs were used as negative controls, and PBMC with 5 *µ*g/ml of phytohemagglutinin (PHA, Sigma-Aldrich, St. Louis, MO) was used as a positive control for cell reactivity. Following incubation, 50-*µ*L aliquots were recovered for the cell viability assay under each experimental condition. Before centrifugation at 400 × *g* for 5 minutes, supernatants were collected, sterile filtered (0.22 *µ*m), and stored at −80°C for further assays.

### 2.8. Determination of Cytokines

The levels of a panel of cytokines (TNF-*α*, IL-1beta, IL-6, IL-8, and IL-10) present in the coculture supernatants were determined using a Cytometric Bead Array (CBA) Human Inflammatory Cytokines Kit (BD, Becton Dickinson, Franklin Lakes, New Jersey, U.S.A.) according to the manufacturer's instructions.

### 2.9. Statistical Analysis

Data were analyzed using GraphPad Prism version 9.0.0 (GraphPad Software, San Diego, California, USA, https://www.graphpad.com) and Python 3.12 (https://www.python.org) for Mac. The distribution of virulence genes among MDR and NMDR strains and their presence in carbapenem-resistant and fluoroquinolone-resistant strains were calculated using Fisher's exact test for each gene. The correlation between virulence genes and resistance rates (fluoroquinolones, carbapenems, and multidrug resistance) and the type of infection were calculated using the DataFrame.corr() method in Python. For the presence of virulence genes in biofilm production, the chi-squared test was used, as well the relationship between virulence genes and the infection involved. Differences observed in PBMC assays between groups (MDR/*exoU*, NMDR/*exoU*, MDR/*exoS*, and NMDR/*exoS*) were analyzed using a nonparametric test of variance (Kruskal–Wallis and Dunn's post-test). Statistical significance was set at *p* < 0.05.

## 3. Results

### 3.1. Sample Collection

As shown in Table 1, seventy-five *Pseudomonas aeruginosa* strains associated with HCAIs were obtained in a year, corresponding to 28 respiratory tract infections, 18 bacteremia, 11 infections from the surgical site, 9 from skin and soft tissue, and 9 from ocular and urogenital infections.

Forty-three patients were males, and thirty-two were female. Most isolates were identified in adults between 36 and 60 years of age (*n* = 27). The mean length of hospital stays was 42.2 days (range, 3–243 days). Most patients were discharged because of clinical improvement (*n* = 59), and only five deaths were associated; however, no statistical difference was found.

### 3.2. Antimicrobial Susceptibility

Among the antibiotics tested (Table 2), different levels of resistance were observed: 24% and 27% were nonsusceptible to amikacin and ciprofloxacin, respectively. Aztreonam showed the highest resistance (48%, *n* = 36), followed by meropenem (43%, *n* = 32). The remaining strains presented approximately 30% nonsusceptibility, and 25 strains were classified as multidrug-resistant.

### 3.3. Virulence Genes in Infections, Biofilm, and Antibiotics

After the PCR analyses, 16 genes were screened. The genes were classified according to the associated infection, biofilm production, susceptibility to fluoroquinolones, carbapenems, and multidrug-resistant status to determine any association or correlation.

**Figure 1:**
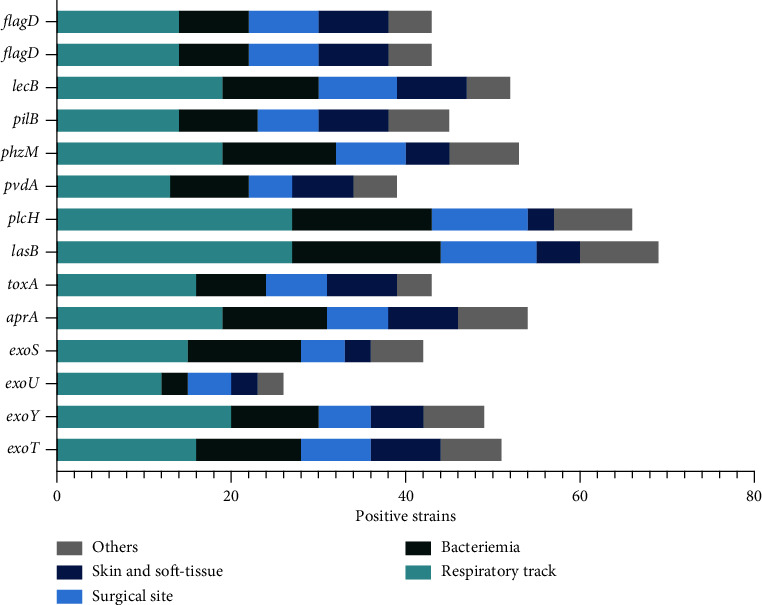
The frequency of virulence genes among *P. aeruginosa* strains according to infection-type comparisons among groups was determined using the chi-square test; ^*∗*^*p* < 0.05 was considered statistically significant.

Classified by the type of infection, the most prevalent genes were *plcH, lasB,* and *algD, as shown in*Figure 1, which were present in at least 90 percent of the strains, followed by *exoT, exoY, aprA, phzM*, and *lecB*, which were present in 60 percent of the strains. However, this difference was not statistically significant.

**Figure 2:**
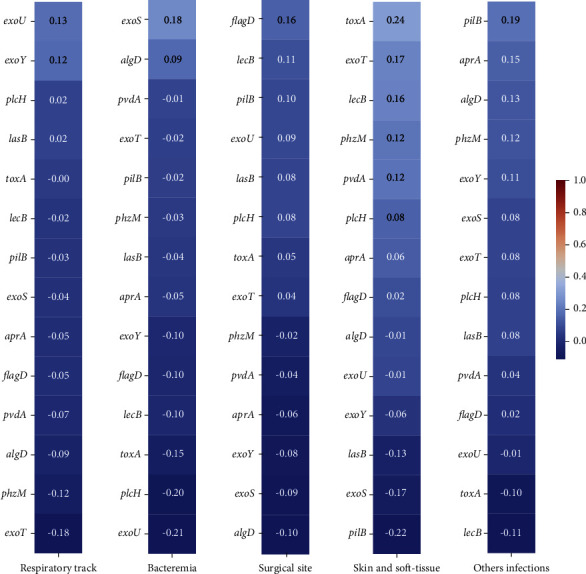
Correlation between the type of infection and virulence genes was calculated using the DataFrame.corr() method in Python.

The correlation between the type of infection and the virulence genes shown in Figure 2, with a slight positive correlation between toxA and skin and soft-tissue infection and a negative correlation between pillB and skin and soft-tissue infection as well as between *exoU* and bacteremia, was detected; nevertheless, any strong positive or negative correlation was found.

As expected, most strains were medium- or high-biofilm producers (Table 3). However, three genes showed significant differences in *exoY* and *toxA,* with the majority being the stronger producers with 29 (38.7%) and (37.3%) strains, respectively. The *lecB* gene was mainly present in moderate producers (27 strains; 36.0%). Interestingly, some trends were observed. *The pilB* and *exoU* genes had a higher presence in medium biofilm producers, whereas the rest of the genes were present in strong producers.

**Figure 3:**
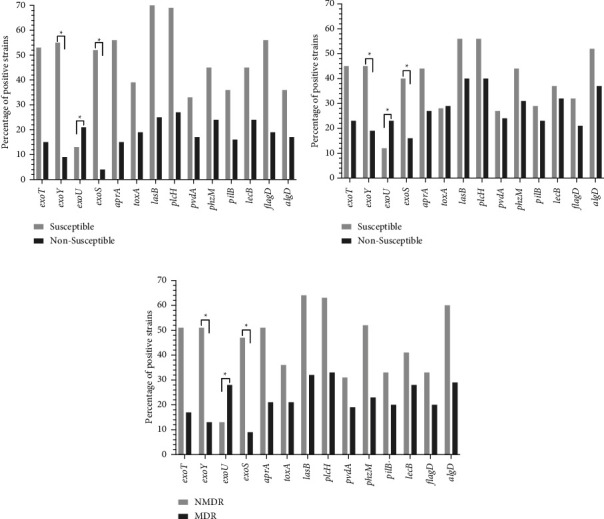
Frequency of the virulence genes between *P. aeruginosa* strains according to their susceptibility to (a) fluoroquinolones, (b) carbapenems, and status as (c) multidrug-resistant. Comparisons between groups were performed using Fisher's exact test; *p* < 0.05 was considered statistically significant.

**Figure 4:**
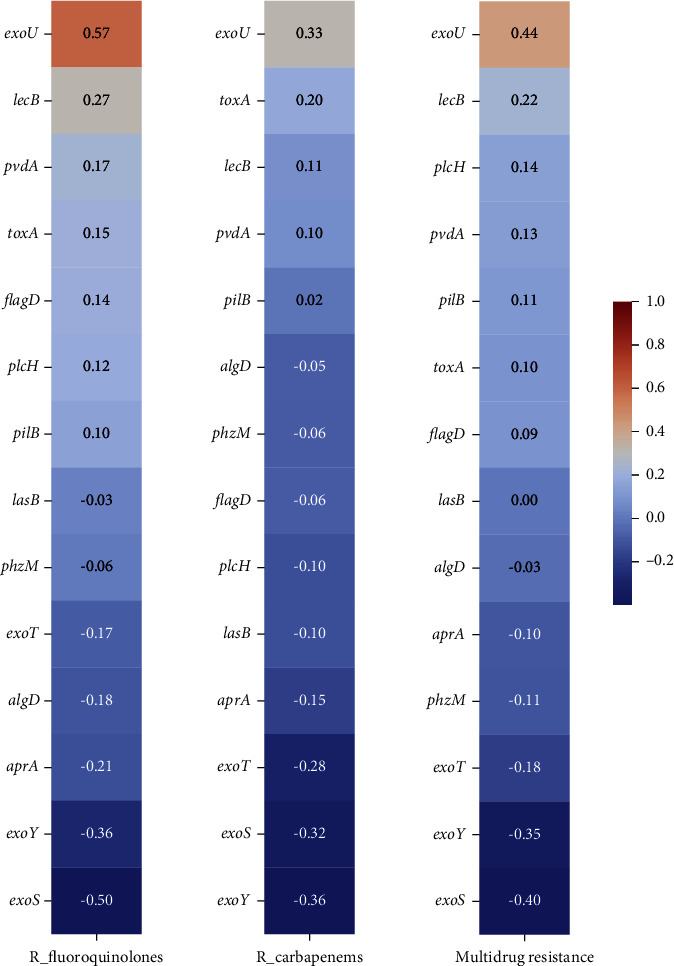
Correlation between virulence genes and resistance rates (fluoroquinolones, carbapenems, and multidrug resistance) was calculated using the DataFrame.corr() method in Python.

Later, it explored how the genes could be distributed among strains with different resistance patterns, and in the case of resistance to carbapenems and quinolones, multidrug resistance was interesting. As shown in Figure 3, statistical differences in two of the genes belonging to SST3, *exoS* and *exoU*, were found. Additionally (Figure 4), a negative correlation between exoS and the different resistance patterns was −0.36 and −0.50 complementary, and *exoU* in the same resistance patterns was positive between 0.33 and 0.57. The last one was relevant because of the association between exotoxin U and strains with nonsusceptibility and MDR and its relationship with cellular destruction.

### 3.4. Cell Viability after Bacterial Coculture and Cytokine Production

**Figure 5:**
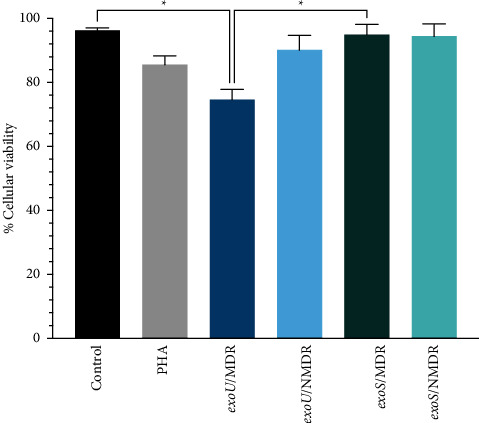
Cellular viability of PBMC incubated with *P. aeruginosa* strains. The mean values ± SD from four independent experiments are shown. The Kruskal–Wallis multiple comparison test was performed; ^*∗*^*p* < 0.05 was considered statistically significant.

Based on the results of the resistance and frequency of genes, four groups were formed, two bearing the *exoU* gene (*exoU*/MDR and *exoU*/NMDR) and two bearing the *exoS* gene (*exoS*/MDR and *exoS*/NMDR). The first assay involved checking the cellular viability (Figure 5), and significant differences were observed. The *exoU*/MDR group had higher mortality rates than the control and *exoS*/NMDR groups.

**Figure 6:**
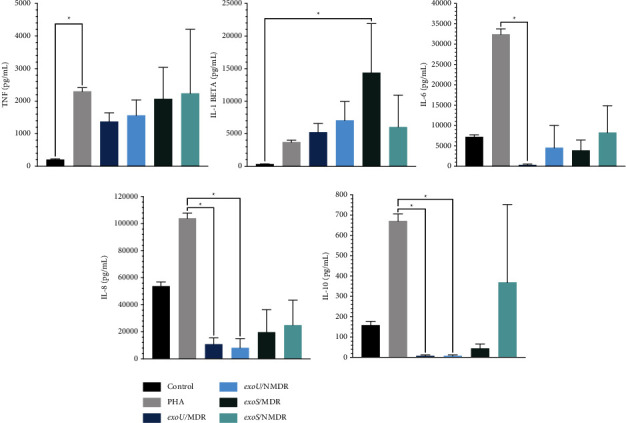
Cytokine profiles of PBMC stimulated with *P. aeruginosa* strains. Cytokine levels were evaluated using a flow cytometric multibead capture assay. The mean values ± SD from four independent experiments are shown. The Kruskal–Wallis multiple comparison test was performed; ^*∗*^*p* value <0.05 was considered statistically significant.

As shown in Figure 6, some differences were observed in cytokine assays. The presence of the strains did not affect the secretion of TNF; however, IL-6, IL-8, and IL-10 showed lower levels of secretion in the *exoU*/MDR group than in the positive control group, and *exoU*/NMDR had lower secretion of IL-8 and IL-10. Interestingly, IL-1beta was higher in *the exoS/MDR group* than in the control group. These findings provide interesting insights into how the presence of *P. aeruginosa* affects the in-vitro profile of cytokines.

## 4. Discussion

*P. aeruginosa* is an opportunistic pathogen considered a public health problem in every country. Despite the attention being focused on antibiotic resistance, this bacterium is much more complex. It possesses intrinsic resistance, a large genome capable of harboring new resistant genes, and biofilm, a barrier that confers its physical protection. In addition, this pathogen carries an extraordinary repertory of virulence factors, which this study aimed to assess.

It is difficult to scrutinize patient demographics in Mexico because there is little data about how *P. aeruginosa* appears in HCAIs, and the most recent descriptive analysis was made by González-Olvera and Cols. in 2019. They found a mean of five strains per month compared with six strains per month in this study, comparing the population density of the municipalities of each study, and found that San Luis Potosi has good management of nosocomial infections (NI). In addition, the predominant type of infection was on the respiratory tract (ten percent more), while for González-Olvera, most of the cases were from the urinary tract. [28–30]. Another study focusing on *P. aeruginosa* was conducted by Elmouaden and Cols. in 2019, with a total of 87 strains classified as NI in two years but not classified according to the type of infection [31]. Aside from these studies to date, no other study has used the same approach as the present study. However, the epidemiological data are concordant, indicating that the highest rates of infection by *P. aeruginosa* are in the respiratory tract [5, 9].

Something remarkable about the strains in this study is the resistance rates detected: 42.7% of the strains in this study were resistant to carbapenems and 33.3% of MDR strains. These rates are part of the increasing global problem; in this respect, many studies have been conducted, but Hojarcada and Cols. resumed this in an excellent review. In some geographical areas, the rates of MDR are between 15% and 30%; in Europe, 13.7% are resistant to at least three antimicrobial groups. In the United States, the situation is not better; MDR pathogens cause 13% of severe HIAs [32]. In Mexico, the data are limited because of the lack of a national network, and some sources suggest that resistance has diminished in the last ten years. The most recent study was conducted by Garza-González in 2019 and did not include information about the state of San Luis Potosi; they found that 27.8% resistance to meropenem was considerably lower than that found in this study [33]. It is worth mentioning that the lack of information about the country's situation has been attributed to problems such as self-prescription, low quality of generic antibiotics, the use of antibiotics in agriculture, and corruption [34].

*P. aeruginosa* is a pathogen with great versatility in virulence, and this study attempted to find a correlation between virulence genes, type of infection, rates of resistance, and biofilm production. In the first respect, nonstatistical differences were found, which may be because, as some authors point out, genetic differentiation or changes in gene expression occur in the transition from an acute to a chronic infection, which leads to the silencing of genes associated with acute infections when they progress to chronicity or biofilm production [35]. Furthermore, at the time of publication of this manuscript, most studies count virulence genes in all strains rather than counting them according to the type of infection; only one study with a classification similar to this one was found; Fazeli in 2014 found wound infection as the most frequent and *exoS* as the most prevalent gene; for this project, respiratory tract infections, *plcH*, and *lasB* genes are the most common ones, mentioning that discrepancies may be due to geographical differences [36].

Because of the behavior of the data and evidence from other studies, it was considered important to detect genes in different biofilm producers. Statistical differences were observed, and *exoY* and *toxA* were prevalent in strong producers, which is not the first report of an association between *exoY* and biofilm producers. Half of the Azimi strains in 2016 (*n* = 150) were producers and harbored *exoY* [37]. Similarly, in 2018, Asadpour found genes encoding exotoxin A in stronger producers, suggesting that biofilm-forming strains are more virulent, potent, and resistant [38]. According to the present study and in agreement with Bogiel T. et al., there may be a potential relationship between virulence factors and biofilm production rates, suggesting that biofilm producers are more virulent [39]. In a completely different scenario, Passos da Silva et al. recently demonstrated that *lecB* coding for a glycoprotein can bind the exopolysaccharide PSL and stabilize the biofilm matrix, which explains why this gene is present in 69.3% (*n* = 52) of their strains [13] and 45.3% (*n* = 49) in this study.

A classic scenario for bacteria is that they are considered simple life forms with a small space in their genome, and some hypothesize that a strain cannot harbor a plethora of virulence factors while containing many resistance-related genes. However, this has changed in recent years; the relationship between antibiotic-resistant genes and virulence factors might follow a Darwinian model, resulting in the emergence of virulent and resistant clones, and precisely, the *P. aeruginosa* large genome is an excellent candidate to acquire both characteristics [40, 41]. Since 2008, twenty-seven papers have reported a relationship between the *exoU* gene and resistance to carbapenems, including reports of community and nosocomial infections [42].

The evidence in this study suggests a possible relationship between virulence genes and resistance rates; nevertheless, there are very few studies looking for this relationship, and some have not found significant differences [37]. Although this study revealed differences, it found strains that harbored the *exoU* gene, were resistant to fluoroquinolones and carbapenems, and were considered MDR. These findings are consistent with the data of Takata et al. and Subedi et al. in 2018, who found that the *exoU-positive* genotype is more frequent in nonsusceptible fluoroquinolones, carbapenems, and MDR strains [24, 43]. In 2019, Horna found that strains resistant to carbapenems and fluoroquinolones and classified as MDR also harbored the *exoU* gene [44]. Kainuma et al. found the same phenomenon and suggested that *exoU* carriers have a greater potential to spread within hospital units, which, if true, represents a major risk [45].

The final aim was to explore a possible relationship among the genes of T3SS, specifically *exoS* and *exoU*, multidrug resistance, and the production of cytokines (TNF, IL-1beta, IL-6, IL-8, and IL-10). The first approach was to determine the effects of these bacteria on cellular viability. As shown in Figure 5, *exoU*/MDR strains presented higher mortality than the other strains owing to their potent phospholipase activity, which causes necroptosis and cell lysis [46], and *the exoS* groups showed a higher survival rate. Some evidence points out that *exoS* may dampen the immune response, provoking cell death by apoptosis, which is slower than necrosis [47]. Information about how *P. aeruginosa* can affect the pattern of cytokine expression is limited; one of the most accurate studies was made by Christopher H. The Moody group related the effect of a recombinant *exoS* with the mRNA of cytokines from PBMC. They found a decrease in IL-10 and an increase in TNF, IL-6, IL-8, and IL-1beta [48, 49]. They worked with recombinant proteins. The present study attempted to evaluate whether these results are transferable to clinical strains, which is partial, and observed a significant increase in IL-1beta with a significant difference in the MDR group. A possible explanation is provided by Grandjean et al., who found that ExoS can convert prointerleukin (IL-1beta) into mature IL-1beta [50]. IL-10 (NMDR group) and TNF (MDR, NMDR group) levels also increased, but the difference was not statistically significant, which could be an indirect effect of ExoS through NF-*κ*B [20].

For the *exoU* group, the first finding was higher levels of IL-1beta, although nonsignificant, than those in the control group. Hardy et al. in 2022 showed that ExoU has a noncytolytic function through the transitory activation of caspase-1 and proIL-1beta. Moreover, interesting findings emerged: IL-6, IL-8, and IL-10 levels decreased, but only in MDR strains. At the time of publication of this manuscript, there were three papers on the relationship between *exoU* and IL-8; one of them addressed the relationship with IL-6, and the other mentioned the effect of this gene on IL-10. All these studies reported that strains with *exoU* caused an increase in the cytokines tested. This discrepancy may be due to the articles being published before 2012 and the use of different cell lines. Today, considering the results of Hardy, a second look is taken at the relationship between these exotoxins and their effects on cytokines [51–54]. However, a limitation in this study is that these results are the product of an in-vitro model, and the results should be interpreted with caution due to the complexity of the study and the possibility that there are intracellular cytokines that could cross the membranes of the cells undergoing cell death and affect the cytokine pattern.

In conclusion, the appearance of the *exoU* virulence gene in MDR strains has increased in recent years, which is concerning because it is an indicator of strains with a more aggressive and difficult-to-treat infectious capacity. More importantly, the fact that these strains present a different cytokine profile than expected represents the possible existence of unknown mechanisms of how these strains interact with host cells, which may represent a benefit for the progression of the infection.

## Figures and Tables

**Table 1:** The clinical and demographic characteristics of patients with *Pseudomonas aeruginosa* infection were included in the study (*n* = 75).

	*n*	%
Sex		
Male	43	57.3
Female	32	42.7
Age (years)		
Infants (0-1)	8	10.7
Children (2–10)	6	8
Adolescents (11–17)	6	8
Young adult (18–35)	15	20
Adults (36–60)	27	36
Senior (>60)	13	17.3
Length of stay (days)		
Mean	42.5	
SD	35.6	
Range	3–243	
Hospital discharge		
Improvement	59	78.7
No associated death	11	14.7
Associated death	5	6.7
Type of infection		
Respiratory tract	28	37.3
Bacteremia	18	24
Surgical site	11	14.7
Skin and soft tissue	9	12
Others	9	12

**Table 2:** Antibiotic susceptibility patterns of *Pseudomonas aeruginosa* causing HIAs (*n* = 75).

Antibiotic	Susceptible *n* (%)	Nonsusceptible *n* (%)
Amikacin	57 (76.0)	18 (24.0)
Cefepime	51 (68.0)	24 (32.0)
Gentamicin	52 (69.3)	23 (30.7)
Piperacillin/Tazobactam	51 (68.0)	24 (32.0)
Aztreonam	39 (52.0)	36 (48.0)
Ciprofloxacin	55 (73.3)	20 (26.7)
Meropenem	43 (57.3)	32 (42.7)

**Table 3:** Relationship between virulence genes of *Pseudomonas aeruginosa* causing HIAs and level of biofilm production (*n* = 75).

Gene associated with virulence factor	Biofilm production		
	Weak *n* (%)	Moderate *n* (%)	Strong *n* (%)
*exoT*	1 (1.3)	24 (32.0)	26 (34.7)
*exoY* ^*∗*^	3 (4.0)	16 (21.3)	29 (38.7)
*exoU*	1 (1.3)	16 (21.3)	9 (12.0)
*exoS*	2 (2.7)	17 (22.7)	23 (30.7)
*aprA*	2 (2.7)	21 (28.0)	30 (40.0)
*toxA* ^*∗*^	0 (0)	15 (20.0)	28 (37.3)
*lasB*	3 (4.0)	33 (44.0)	36 (48.0)
*plcH*	3 (4.0)	33 (44.0)	36 (48.0)
*pvdA*	1 (1.3)	18 (24.0)	19 (25.3)
*phzM*	1 (1.3)	23 (30.7)	32 (42.7)
*pilB*	2 (2.7)	20 (26.7)	17 (22.7)
*lecB* ^*∗*^	3 (4.0)	27 (36.0)	22 (29.3)
*flagD*	1 (1.3)	18 (24.0)	21 (28.0)
*algD*	2 (2.7)	28 (37.3)	37 (49.3)

Comparisons between groups were performed using the chi-square test; ^*∗*^*p* < 0.05 was considered statistically significant.

## Data Availability

The data used to support the findings of this study are restricted by the ethics board of the hospital “Dr. Ignacio Morones Prieto” in order to protect patient privacy. Data are available from the corresponding author to this research, Perla Niño-Moreno (ncarmenp@uaslp.mx), for researchers who meet the criteria for access to confidential data.

## Abbreviations

HAIs: Healthcare-associated infections
PBMC: Peripheral blood mononuclear cells
T3SS: Type three secretion system
MDR: Multidrug-resistant
WHO: World Health Organization
SSI: Surgical site infection.

## Supplementary Materials

Supplementary Materials Consist of a list of the primers used in our research, including the sequence, the temperature of melting, and the length of the product of PCR expected for each gene included in the research.
