# Supplementary material for: Interesting Cytokine Profile Caused by Clinical Strains of Pseudomonas aeruginosa MDR Carrying the exoU Gene
Source: Int J Microbiol. 2024 Jun 30;2024:2748842. doi: 10.1155/2024/2748842 (PMC11227949; doi:10.1155/2024/2748842)
Supplement: Supplementary Materials — Consist of a list of the primers used in our research, including the sequence, the temperature of melting, and the length of the product of PCR expected for each gene included in the research. [file 2748842.f1.docx]

Supplementary table 1. Consist in a list of the primers used in our research, including the sequence, the temperature of melting and the length of the product of PCR expected for each gene included in the research.

| Gene | Primer | | Tm (Cº) | Expected Amplicons (pb) |
| --- | --- | --- | --- | --- |
| *pilB* | F | ATG AAC GAC AGC ATC CAA CT | 58 | 826 |
|  | R | GGG TGT TGA CGC GAA AGT CGA T |  |  |
| *algD* | F | ATG CGA ATC AGC ATC TTT GGT | 60 | 1311 |
|  | R | CTA CCA GCA GAT GCC CTC GGC |  |  |
| *flag* | F | TTA GCG CAG CAG GCT CAG AAC | 68 | 1185 |
|  | R | ATG GCC TTG ACC GTC AAC AC |  |  |
| *lasB* | F | GGA ATG AAC GAG GCG TTC TC | 61 | 300 |
|  | R | GGT CCA GTA GTA GCG GTT GG |  |  |
| *aprA* | F | TGTCCAGCAATTCTCTTGC | 51 | 1017 |
|  | R | CGT TTT CCA CGG TGA CC |  |  |
| *plcH* | F | GAA GCC ATG GGC TAC TTC AA | 58 | 307 |
|  | R | AGA GTG ACG AGG AGC GGT AG |  |  |
| *toxA* | F | CTG CGC GGG TCT ATG TGC C | 61 | 352 |
|  | R | GAT GCT GGA CGG GTC GAG |  |  |
| *phzM* | F | GGA TCG ACA GCG ACG AGA CG | 61 | 194 |
|  | R | CTC GCC GTA GAA CAG CAC CAT |  |  |
| *exoS* | F | CTT GAA GGG ACT CGA CAA GG | 58 | 504 |
|  | R | TTC AGG TCC GCG TAG TGA AT |  |  |
| *exoU* | F | GGG AAT ACT TTC CGG GAA GTT | 58 | 428 |
|  | R | CGA TCT CGC TGC TAA TGT GTT |  |  |
| *exoT* | F | CAA TCA TCT CAG CAG AAC CC | 54 | 1159 |
|  | R | TGT CGT AGA GGA TCT CCT G |  |  |
| *exoY* | F | TAT CGA CGG TCA TCG TCA GGT | 59 | 1035 |
|  | R | TTG ATG CAC TCG ACC AGC AAG |  |  |
| *pvdA* | F | GAC TCA GGC AAC TGC AAC | 58 | 1281 |
|  | R | TTC AGG TGC TGG TAC AGG |  |  |
| *lecB* | F | GGC AAC ACA AGG AGT GTT CA | 60 | 112 |
|  | R | GAC GGT TTC GTT GTT GAC CT |  |  |
